# Supplementary figures and images for: Sex ratios in flux: seasonal dynamics and methodological insights in Rumex species
Source: PeerJ. 2025 Dec 9;13:e20391. doi: 10.7717/peerj.20391 (PMC12700118; doi:10.7717/peerj.20391)

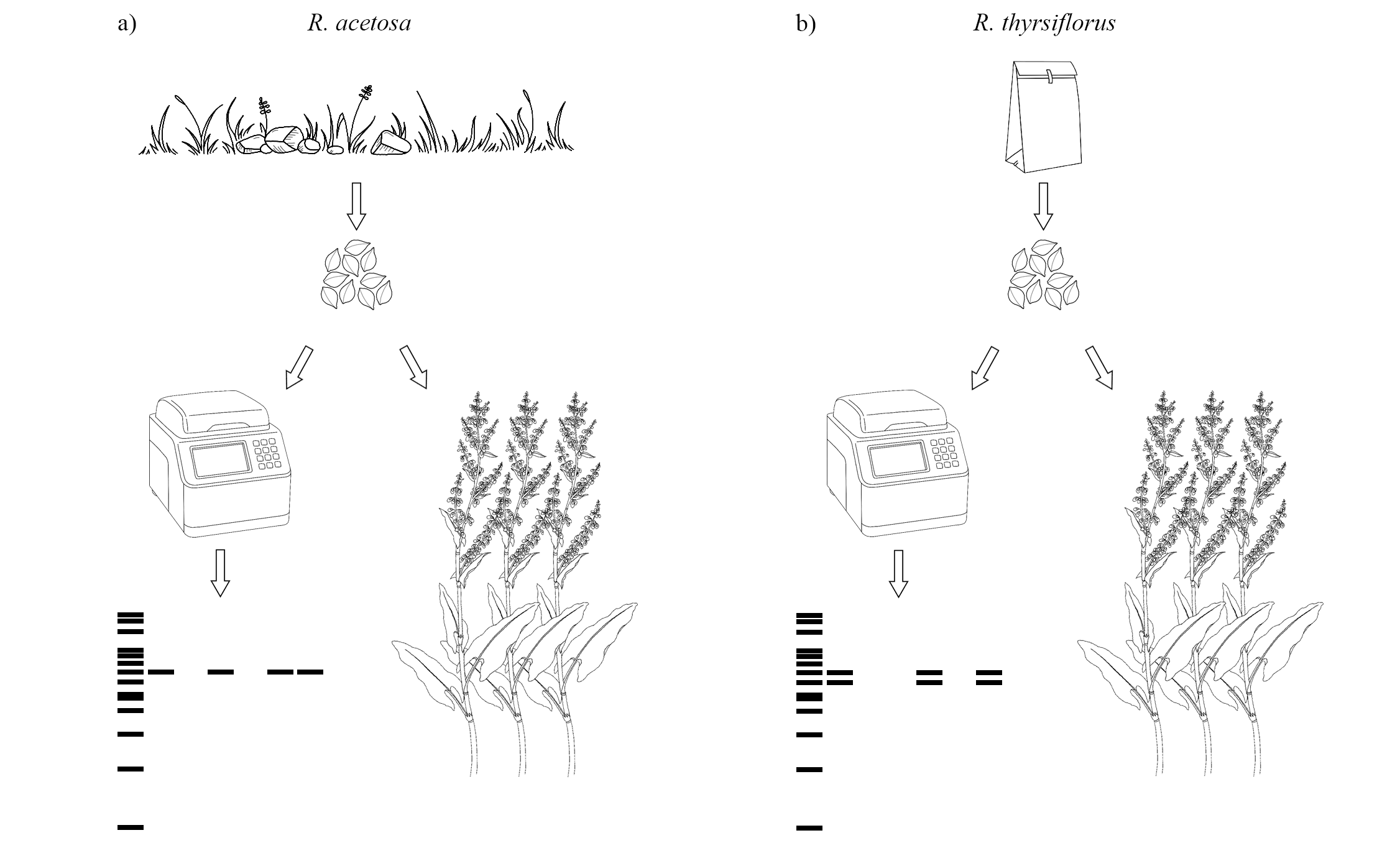

Supplement: Supplemental Information 8 — Seeds of Rumex acetosa and R. thyrsiflorus were randomly selected from seed pools. One part of the seeds was analyzed using molecular methods (PCR, electrophoresis) for sex determination (male individuals show one or two marker bands, while female individuals show no bands). The remaining seeds from the same pools were used for sowing in the field experiment. The differences between species involved both seed origin and PCR results for male individuals: (a) R. acetosa –seeds were collected from plants growing in natural populations; male individuals showed one marker band. (b) R. thyrsiflorus–seeds were purchased commercially; male individuals showed two marker bands. [file peerj-13-20391-s008.png]

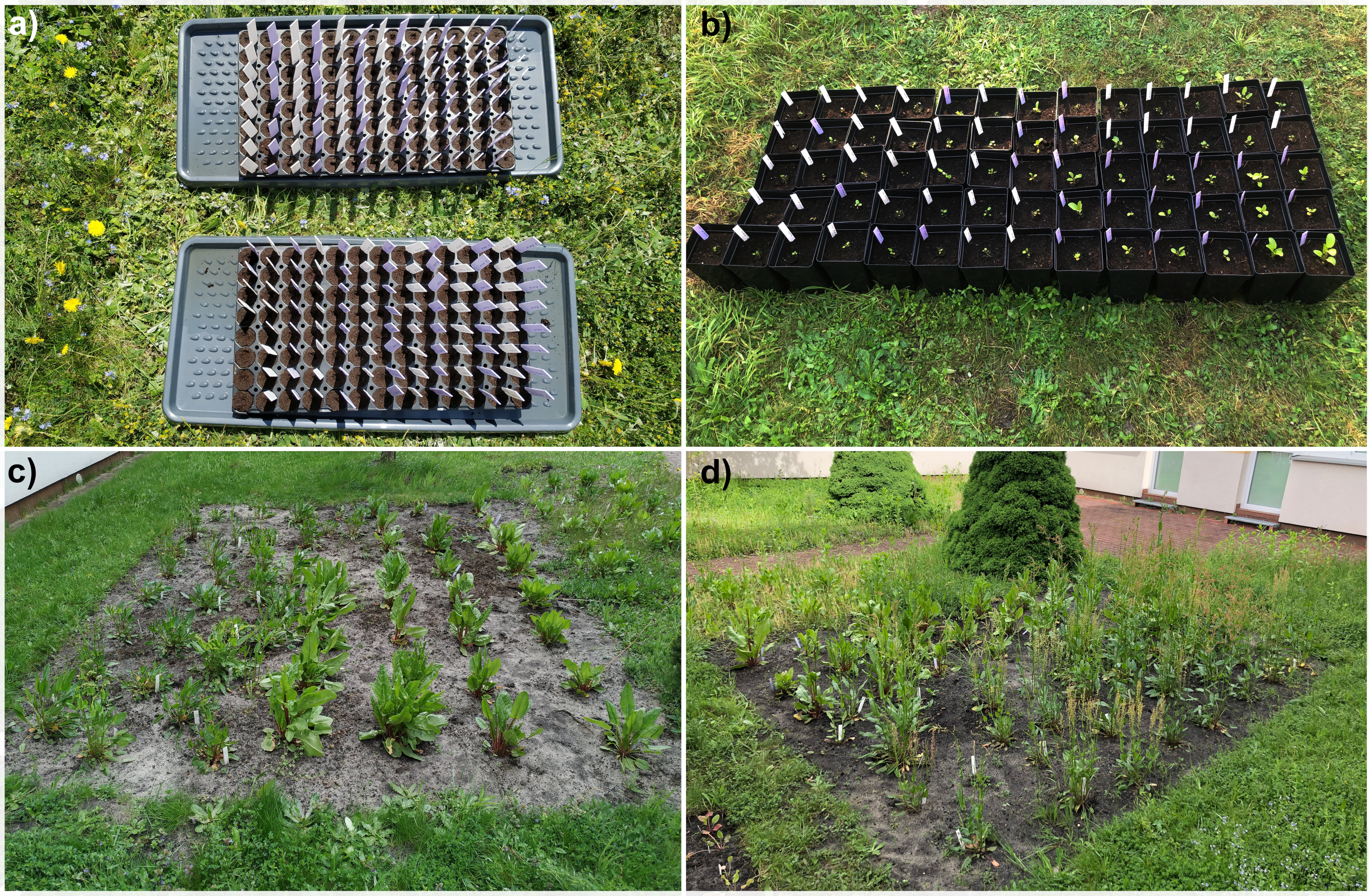

Supplement: Supplemental Information 9 — a) seeds in peat discs in pots; b) young seedlings in pots; c) plants growing in the soil; d) sexually mature individuals. [file peerj-13-20391-s009.png]

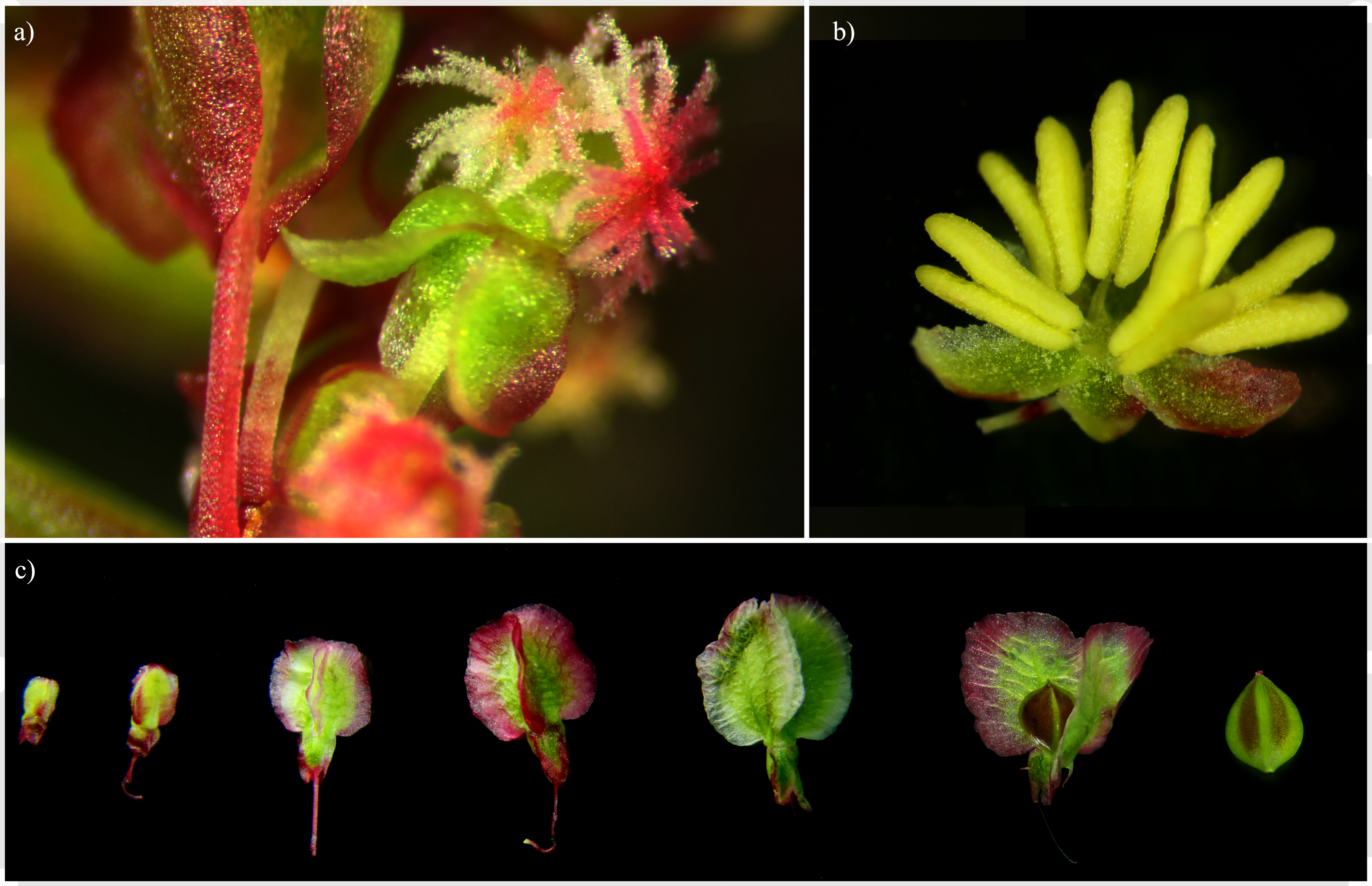

Supplement: Supplemental Information 10 — In both species examined, flowers are morphologically similar; in Rumex acetosa, they can be slightly more yellowish, while in R. thyrsiflorus, they are often reddish. a) female flower of R. thyrsiflorus with pistils; b) male flower of R. thyrsiflorus with pollinating anthers; c) development of a pollinated female flower of R. thyrsiflorus - from a young fruit to a seed. [file peerj-13-20391-s010.png]

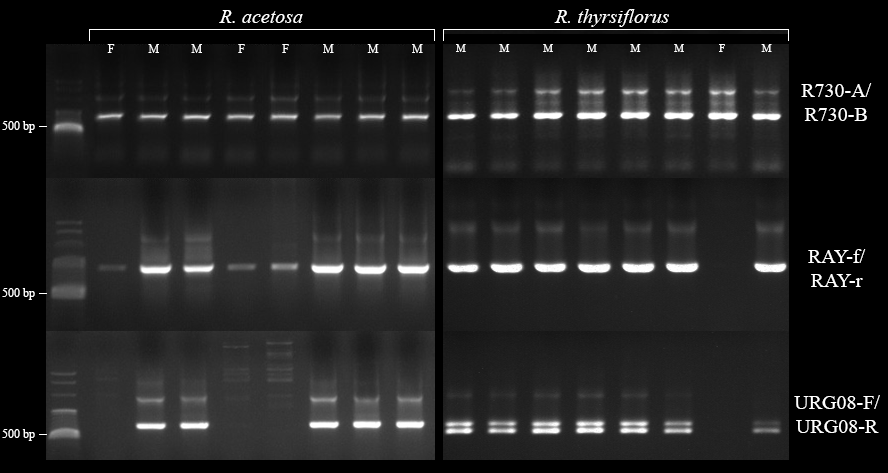

Supplement: Supplemental Information 11 — R730 primers verified DNA quality. RAY primers distinguished male individuals (stronger bands in males). URG08 primers confirmed sex (bands in male, absent in female individuals) and identified species (one band in R. acetosa males, two bands in R. thyrsiflorus males). L, ladder; M, male individuals; F, female individuals. [file peerj-13-20391-s011.png]
